# Human Astrovirus Gastroenteritis in Children, Madagascar, 2004–2005

**DOI:** 10.3201/eid1405.070563

**Published:** 2008-05

**Authors:** Dimitrios C. Papaventsis, Winifred Dove, Nigel A. Cunliffe, Osamu Nakagomi, Patrice Combe, Pierre Grosjean, C. Anthony Hart

**Affiliations:** * University of Liverpool, Liverpool, UK; † Nagasaki University, Nagasaki, Japan; and ‡Institut Pasteur, Antananarivo, Madagascar; 1 Deceased.

**Keywords:** Astrovirus, genogroup, child, Madagascar, dispatch

## Abstract

We report data regarding the molecular epidemiology of human astrovirus (HAstV) infections among children in Madagascar. In a 13-month study, 5 HAstV isolates were detected in fecal samples from 237 children (2.1%) by reverse transcription–PCR. Phylogenetic analysis showed the cocirculation of usual and unusual HAstVs.

Human astroviruses (HAstVs) belong to the *Astroviridae* family, which is divided into 2 genera: *Mamastrovirus* (mammalian viruses) and *Avastrovirus* (avian viruses) (*1*). They are single-stranded positive-sense RNA viruses with a characteristic 5- or 6-pointed star, which has an electron-dense center when viewed by negative-stain electron microscopy. Their genome is 6.8–7.3 kb in length and includes 3 overlapping open reading frames (ORFs) (*2*). ORF1a and ORF1b encode nonstructural proteins (serine protease and RNA polymerase, respectively). ORF2 encodes the capsid precursor. There are 8 serotypes of HAstVs; type 1 is the most prevalent worldwide (*3*).

Molecular assays such as reverse transcription–PCR (RT-PCR) and molecular typing methods have advanced our understanding of HAstV epidemiology. Today, astroviruses, along with rotaviruses and caliciviruses, are considered important viral agents of acute pediatric gastroenteritis (*2*). These viruses have been associated with endemic diarrheal episodes and outbreaks of gastroenteritis in industrialized (*4*,*5*) and nonindustrialized countries (*6*,*7*). Although Madagascar is a society of considerable diversity, no studies have yet been reported on the prevalence and molecular epidemiology of HAstV among children with acute gastroenteritis in the country.

## The Study

From May 2004 through May 2005, a study of acute gastroenteritis in children (newborn to 16 years) was undertaken by Institute Pasteur, Antananarivo, Madagascar. This study was approved by the Ethical Review Board of Institut Pasteur, Madagascar, and the National Ethical Committee of Madagascar. It was funded internally by the University of Liverpool. Antananarivo is the capital city of Madagascar with a population of ≈4 million persons. Fecal samples were collected from children with acute dehydrating gastroenteritis who had been brought to the rehydration clinics and hospitals of Antananarivo for treatment; samples were stored at –80°C until they were analyzed at the University of Liverpool, United Kingdom.

All samples were screened by RT-PCR for astrovirus and norovirus and by ELISA with subsequent genotyping for rotavirus, as described (*8*–*11*). In brief, viral RNA was extracted from 150 μL of 10%–20% fecal suspensions in phosphate-buffered saline by using a guanidine and silica method (*12*) and reverse transcribed by using random hexamers (Sigma-Genosys, Dorset, UK). Primers Mon244/245 and Mon269/270 were then used to amplify a 413-bp and a 449-bp fragment of ORF2, respectively (*8*). If no amplicon was obtained, primers Mon 340/348 were used to amplify a 289-bp fragment of ORF1a. Amplification products were purified by using Minispin columns (Amersham, Buckinghamshire, UK) and sequenced by Cogenics Lark Technologies (Hope End, Essex, UK).

Phylogenetic relationships were examined by using the ClustalW multiple alignment program (EMBL, Heidelberg, Germany). Phylogenetic trees were constructed according to the neighbor-joining method with ClustalX (version 1.83); the alignment file obtained by analysis with ClustalW bootstrap values on a scale from 1 to 1,000 was also calculated. Unrooted phylograms of HAstV isolates from the present report and reference strains were plotted in the PHYLIP format output by using the TreeView software version 3.0 (http://taxonomy.zoology.gla.ac.uk/rod/treeview.html). Assignment of HAstV to genotype was done according to the scheme proposed by Belliot et al. (*9*). The nucleotide sequences of the Malagasy strains have been deposited at the GenBank database under accession nos. EF490425–EF490429 and EF519312.

During this 13-month study, 237 children (142 boys and 95 girls) were screened for HAstV infection. Overall, 85% of the children were <3 years, 77% were <2 years, 43% were <1 year, and 3% were newborns. The median age of the study population was 20 months (range 1 day–16 years); for children with astrovirus infection, the median age was 10 months (5–21 months). No infections were recorded among newborns.

No RT-PCR–confirmed isolate was detected by electron microscopy. No HAstVs were detected by using primers Mon269/270. One positive sample was found with primers Mon244/245. This sample and an additional 4 strains produced amplicons with primers Mon340/348. Each isolate was typed successfully by nucleotide sequencing of either partial ORF1a or ORF2 products obtained by using primers Mon340/348 (n = 5) and Mon244/245 (n = 1), respectively (Table).

**Table:** Detection of human astrovirus in fecal specimens from children of Antananarivo, Madagascar, 2004–2005*

Sample no.	ID no. (GenBank accession no.)	Sample date	Patient age, mo/sex	Norovirus PCR	Rotavirus PCR	Astrovirus				
PCR 340/348	PCR 244/245	PCR 269/270	Sequence typing	
			ORF1a	ORF2
1	DT1004 (EF490429)	2004 Jun	12/M	Neg	G2P[4]	Pos	Neg	Neg	Type 1	–
2	DG6013 (EF490425)	2004 Jun	10/F	Neg	Neg	Pos	Neg	Neg	Type 3	–
3	DR0034 (EF490427)	2004 Nov	10/M	Neg	Neg	Pos	Neg	Neg	Type 8	–
4	DR0038 (EF490428, EF519312)	2004 Nov	5/M	Neg	G2P[4]	Pos	Pos	Neg	Type 8	Type 2
5	DR0075 (EF490426)	2005 Feb	21/M	Neg	Neg	Pos	Neg	Neg	Type 3	–

*ID, identification; ORF, open reading frame; Neg, negative; Pos, positive; –, not amplified.

In total, 5 HAstVs were detected in 237 children (2.1%). HAstVs were detected throughout the year. Two co-infections with G2P[4] rotavirus were confirmed by RT-PCR. No co-infection with norovirus was found (Table). Sequence analysis of the ORF1a region showed that isolates from Madagascar clustered within the genogroup A strains as described by Belliot et al. (*9*). In particular, 1 strain (DT1004_Madagascar2004_EF490429) grouped with genotype 1, a total of 2 strains (DG6013_Madagascar2004_EF490425 and DR0075_Madagascar2005_EF490426) grouped with genotype 3, and notably, 2 strains (DR0034_Madagascar2004_EF490427 and DR0038_Madagascar2004_EF490428) grouped with the emerging genotype 8. Phylogenetic analysis of the ORF2 region showed that the 1 amplifiable isolate from Madagascar (DR0038_Madagascar2004_EF519312), which was clustered as genotype 8 at the ORF1a region, could now be classified as genotype 2 (Figure).

**Figure:**
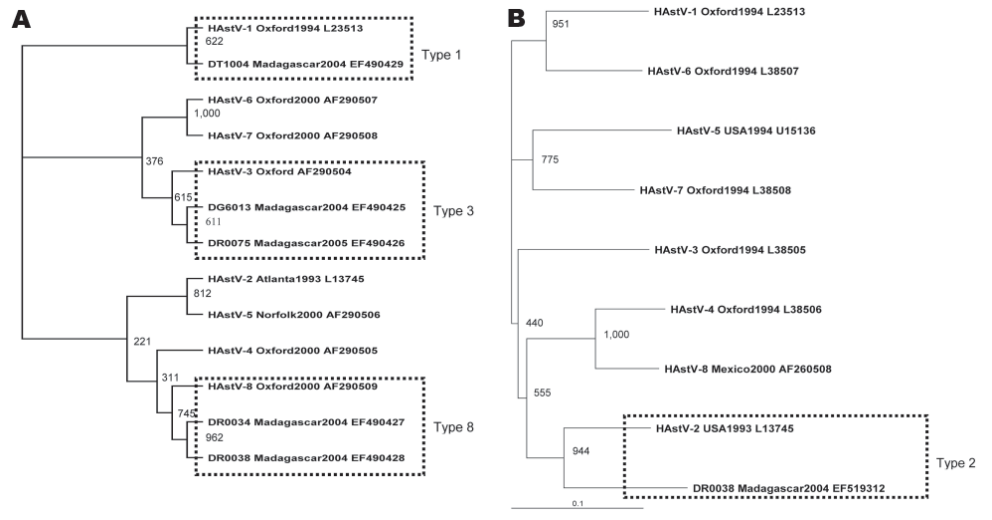
Phylogenetic tree of human astrovirus (HAstV) based on the 289-base region of the open reading frame (ORF) 1a gene (A) and the 413-base region of the ORF2 gene (B). We included 6 novel sequences designated according to isolate code_place/year_GenBank accession no. A) DG6013_Madagascar2004_EF490425, DR0075_Madagascar2005_EF490426, DR0034_Madagascar2004_EF490427, DR0038_Madagascar2004_EF490428, DT1004_Madagascar2004_EF490429; B) DR0038_Madagascar2004_EF519312. We also included 16 sequences of reference astrovirus strains obtained from GenBank, designated according to HAstV genotype_place/year_GenBank accession no.: A) HAstV-1_Oxford1994_L23513, HAstV-2_Atlanta1993_L13745, HAstV-3_Oxford_AF290504, HAstV-4_Oxford2000_AF290505, HAstV-5_Norfolk2000_AF290506, HAstV-6_Oxford2000_AF290507, HAstV-7_Oxford2000_AF290508, HAstV-8_Oxford2000_AF290509; B) HAstV-1_Oxford1994_L23513, HAstV-2_USA1993_ L13745, HAstV-3_Oxford1994_L38505, HAstV-4_Oxford1994_L38506, HAstV-5_USA1994_U15136, HAstV-6_Oxford1994_L38507, HAstV-7_Oxford1994_L38508, HAstV-8_Mexico2000_AF260508. Bootstrap values based on 1,000 generated trees are displayed at the nodes.

## Conclusions

This study systematically examined the role of HAstV in acute gastroenteritis in Madagascar. In a 13-month study, we detected HAstV by RT-PCR in 2.1% of children with gastroenteritis in Antananarivo. This finding establishes HAstV as the third most commonly detected enteric virus in this population, after rotavirus (38%) and norovirus (6%) (*11*). These findings agree with those of previous studies that reported astrovirus infection rates of 1.5% to as high as 26% (*13*,*14*).

The median age of children with HAstV infection was 10 months (range 5–21 months), equivalent to that of the rotavirus-infected group (median age 10 months; range 1 day to 48 months) but lower than that of the norovirus-infected group (median age 18 months, range 3 to 51 months) (*11*). HAstVs were detected in June and November 2004 and February 2005 (2 isolates during each of June and November 2004 and 1 isolate in February 2005). However, because of the small number of isolates detected, we could not determine whether the pattern of HAstV infections in Madagascar was seasonal.

Compared with electron microscopy, new molecular detection and typing methods have greatly enhanced our ability to study the endemic circulation of enteric viruses. Our data indicate that HAstVs are important agents of acute gastroenteritis among children <24 months in Madagascar and that simultaneous circulation of multiple astrovirus genotypes is not rare. Although HAstV-1 has been reported to be the most prevalent type detected worldwide, the presence of HAstV-8 among our isolates indicates the continued worldwide emergence of this unusual astrovirus strain. In addition, phylogenetic analysis of the serine protease and capsid regions of our HAstV strains provided contradictory genotyping results; RNA recombinations may have contributed to significant genomic rearrangements (*15*). Further analysis is needed to confirm our preliminary findings and to investigate the importance of HAstV infections among children in Madagascar.
